# Abnormal neural network connectivity in heart failure with reduced and mild-ranged ejection fraction patients: an independent component and dynamic functional network connectivity analysis

**DOI:** 10.3389/fneur.2025.1626961

**Published:** 2025-10-27

**Authors:** Qian Gao, Junyan Wen, Yi Lu, Jun Li, Baotong Hua, Yin Mo, Yanfei Mao, Yunyun Xu, Ping Xia, Kaipeng Xie, Yizhen Zeng, Ge Wen

**Affiliations:** ^1^Department of Imaging, Nanfang Hospital, Southern Medical University, Guangzhou, China; ^2^Department of Radiology, The 1st Affiliated Hospital of Kunming Medical University, Kunming, China

**Keywords:** heart failure, independent component analysis, dynamic functional network connectivity, functional connectivity, functional magnetic resonance imaging

## Abstract

**Introduction:**

Heart failure (HF) is frequently accompanied by cognitive and affective impairments, yet the neural mechanisms underlying these comorbidities remain insufficiently understood. This study aimed to investigate alterations in static and dynamic functional connectivity (FC) within large-scale brain networks in patients with reduced (HFrEF) and mid-range (HFmrEF) ejection fraction.

**Methods:**

Independent component analysis (ICA) was used to identify resting-state networks (RSNs) and FC disparities between HF patients and healthy controls (HCs) within the RSNs. The ICA, sliding window approach, and k-means clustering analysis were used to compute dynamic functional network connectivity (dFNC) matrices and estimate different dynamic connection states. The temporal characteristics of the two groups were analyzed in each state. The correlations among significantly diverse temporal aspects and clinical measures were finally determined.

**Results:**

Compared to HCs, HF patients showed reduced FC in the right inferior parietal lobule (IPL) within the dorsal attention and frontoparietal networks, alongside increased FC in the salience network. dFNC analysis revealed five recurrent connectivity states. Notably, HF patients exhibited shorter dwell time in a sensory–cognitive segregation state (State 5), and dwell time in this state correlated positively with both left ventricular ejection fraction (LVEF) and Mini-Mental State Examination (MMSE) scores.

**Conclusion:**

The disrupted static and dynamic connectivity in HF patients—marked by alterations in frontoparietal, attention, and salience networks and reduced stability of a sensory–cognitive segregation state—may underlie cognitive and affective vulnerability, providing potential imaging markers for early risk monitoring and management in HF.

## 1 Introduction

Improvements in medical treatment and increased life expectancy have significantly enhanced survival in individuals with cardiovascular diseases, leading to a rising prevalence of chronic heart failure (HF) and its associated comorbidities (1). Neurological comorbidities, such as depression, anxiety and cognitive impairment, tend to be common among HF patients, with the prevalence rate of such conditions being markedly higher compared with the general population (2–5). Approximately 30% and up to 75% of HF patients experience depression and cognitive impairment, respectively (4, 6–8). HF patients with neurological comorbidities often display poor adherence to therapy and a loss of functional independence while experiencing reduced quality of life or early death (9, 10). However, the pathophysiological mechanisms for neurological comorbidities in HF remain unclear. Further exploration in HF patients is needed to provide new imaging evidence for the heart-brain axis theory, better understand the clinical manifestations, and offer appropriate treatment options for these patients.

A common data-driven technique for blind source separation is Independent Component Analysis (ICA). This technique has significantly helped better understand the intrinsic networks within the brain and the functional connectivity (FC) at the network level (11). To investigate dynamic changes in these networks, ICA can be combined with a sliding window approach to estimate time-resolved fluctuations in FC and to identify recurrent connectivity states across windows. This combined framework, known as dynamic functional network connectivity (dFNC), enables the characterization of temporal variability that is not captured by static FC analyses (12). Although the validity of sliding window analysis (SWA) has been questioned (13), studies have shown that meaningful brain states can be identified from short resting-state segments (14, 15). Thus, SWA remains a useful approach, especially in pathological populations where transient FC alterations are expected.

**Figure 1:**
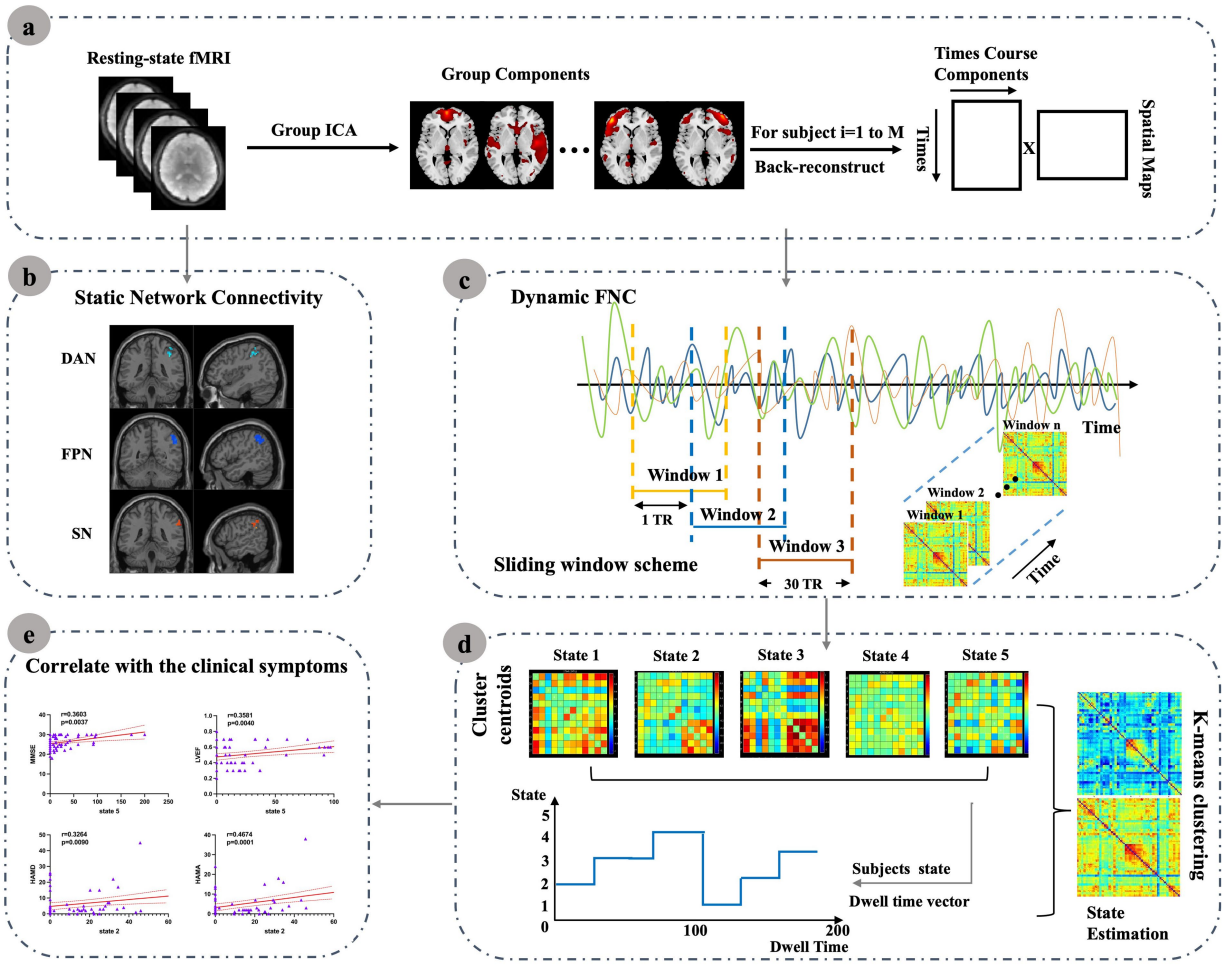
Resting-state fMRI data were preprocessed and submitted to group ICA to extract resting-state networks (RSNs). Static network connectivity was then assessed to characterize internetwork functional alterations in HF patients. For dynamic FNC (dFNC), a sliding-window approach was applied; the resulting time-resolved connectivity matrices were clustered using k-means to derive recurring connectivity states. For each state, temporal characteristics such as mean dwell time and occurrence rate were computed and further correlated with clinical measures. **(a)** Resting-state fMRI data were preprocessed and submitted to group ICA to extract resting-state networks (RSNs). (b) Static network connectivity was then assessed to characterize internetwork functional alterations in HF patients. (c) For dynamic FNC (dFNC), a sliding-window approach was applied; (d) The resulting time-resolved connectivity matrices were clustered using k-means to derive recurring connectivity states. For each state, (e) Temporal characteristics such as mean dwell time and occurrence rate were computed and further correlated with clinical measures.

Neuroimaging evidence has linked HF to structural, functional, and metabolic brain alterations (16–19), which have in turn been associated with cognitive, emotional, and pain-related functions (20). Recent studies in heart failure with preserved ejection fraction (HFpEF) patients have also reported alterations in both static and dynamic functional network connectivity, with changes involving major large-scale networks and associations with cardiac function (21). However, the temporal properties of intrinsic connectivity networks (ICNs) remain poorly characterized in patients with reduced and mid-range ejection fraction (HFrEF and HFmrEF), who often show more pronounced cognitive vulnerability. Static functional connectivity provides only a time-averaged view of brain interactions, which may obscure transient fluctuations and dynamic reconfigurations that are critical for adaptive cognitive and emotional processes (22). In contrast, dynamic functional network connectivity (dFNC) captures short-term variability and recurrent connectivity states, thereby offering unique insights into network instability and impaired brain–heart interactions in HF. To our knowledge, this study is among the first to integrate ICA with dFNC in patients with reduced and mid-range ejection fraction, thereby enabling a joint characterization of static network alterations and time-varying connectivity dynamics. Building on this framework, we further examined whether these network alterations were associated with cardiac function, cognitive performance, and affective symptoms. This study is designed to provide novel insights into how HF disrupts intrinsic brain networks and their temporal dynamics, with potential implications for understanding HF-related brain dysfunction and improving patient management. The overall analytic workflow is summarized in Figure 1.

## 2 Methods

### 2.1 Participant selection

Following the ESC criteria, HF patients were defined in this investigation. Patients with HFmrEF and HFrEF were among those whose ejection fraction was less than 50% (23). HFrEF was characterized by clinically diagnosed HF with an LVEF of < 40%, as determined by echocardiography. HFmrEF referred patients with an LVEF between 40 and 49%, representing a “grey area” of heart function. Participants with contraindications to MRI, history of head trauma, drug or alcohol abuse, or severe physical or neurological deficits were not enrolled. Among those scanned, individuals were further excluded if they showed substandard image quality, ischemic cerebral lesions, or brain tumors. Using this procedure, our prospective HFrEF and HFmrEF cohort initially recruited 44 patients between January 2022 and December 2023. We excluded 7 patients due to ischemic cerebral lesions and 2 patients due to motion artifacts (mean framewise displacement > 0.5 mm, >2.0 mm translation, or >2.0° rotation). Finally, 35 patients were analyzed in the present study. In addition, 28 healthy controls (HCs) were randomly selected from individuals undergoing brain MRI during the same period, after stratification to match the HF group by age, sex, and years of education. Exclusion criteria included a family history of mental illness or the presence of cardiac or psychiatric disorders. and the same additional criteria as for the patient group were applied. All participants were determined to be right-handed based on the Edinburgh Habitual Handedness Scale results. Within 24 h of the MRI scan, they were evaluated employing the Mini-Mental State Examination (MMSE), the 24-item Hamilton Depression Rating Scale (HAMD-24), and the Hamilton Anxiety Rating Scale (HAMA). Table 1 provides information about the participants’ clinical and demographic characteristics.

**Table 1:** Demographic, clinical data, and behavioral measures.

Variables	Heart failure patients (*n* = 35)	Healthy controls (*n* = 28)	χ^2^/t/z	*p*-value
Gender (M/F)	26/9	17/11	1.322	0.250
Age (years)	55.82 ± 10.82	54.92 ± 7.75	−0.370	0.712
Education (years)	9.71 ± 4.56	8.96 ± 4.03	−0.862	0.498
Diabetes (absence/presence)	30/5	25/3	0.179	0.672
Hypertension (absence/presence)	18/17	21/7	3.665	0.056
LVEF (%)	39 ± 8.3	65 ± 6.4	13.33	<0.001
BNP (pg/ml)	1237.19 ± 1423.25	NA	NA	NA
HAMA	7.88 ± 0.42	1.35 ± 0.95	−9.133	<0.001
HAMD	10.00 ± 10.02	1.53 ± 1.07	−8.817	<0.001
MMSE	25.03 ± 3.33	27.10 ± 2.96	2.58	0.011

Two-sample *t*-tests for normalized data, Mann–Whitney U-tests for non-normalized data (HAMA and HAMD scores), and Pearson’s Chi-square test for gender, presence of diabetes and hypertension; LVEF, Left Ventricular Ejection Fraction; BNP, B-type Natriuretic Peptide; HAMA, Hamilton Anxiety Rating Scale; HAMD, Hamilton Depression Rating Scale, MMSE, Mini-Mental State Examination.

### 2.2 Data acquisition and preprocessing

Brain MRI scans were conducted using a GE Discovery 750w 3.0 T scanner. The following echo-planer imaging sequence was then employed to gather fMRI data in the resting state: flip angle = 90°, slice thickness = 3.0 mm, number of slices = 36, field of view = 22.4 × 22.4, TE = 30 ms, TR = 2000 ms, matrix size = 64 × 64, voxel size = 3.0 × 3.5 × 3.5 mm^3^.

Resting-state fMRI data were preprocessed using the DPABI (Data Processing & Analysis of Brain Imaging) toolbox.^1^ Preprocessing included slice timing correction, realignment, normalization to MNI space, detrending, nuisance regression (white matter, CSF, and motion parameters), global signal regression, temporal band-pass filtering (0.01–0.08 Hz), and spatial smoothing with a 6-mm FWHM Gaussian kernel.

### 2.3 ICA and determination of RSNs

Group ICA analysis was performed separately for the HF and HC groups using Version 4.0 of the GIFT toolbox (available at https://trendscenter.org/software/gift/), which decomposed the data into independent components (ICs) that are estimated based on subject-specific spatial maps and time courses across subjects. The data’s dimensionality was initially reduced using principal component analysis (PCA) before determining the number of ICs using the minimum description length (MDL) criteria. Through this approach, 28 and 41 components were identified for the control group and the HF patients, respectively, and these were deemed sufficient for capturing the major large-scale resting-state networks. Independent components were then repeatedly estimated 20 times with the infomax algorithm in ICASSO, with the components subsequently clustered to assess the extent to which the decomposition process was reliable. The group ICA back-reconstruction method was employed to reconstruct each individual’s spatial maps and time courses. By examining the correlations between the spatial maps and established RSN templates, RSN components were automatically identified for each participant. These templates represented brain regions that are commonly associated with RSNs.

A total of ten relevant RSNs were detected. The networks included are the visual network (VIN), somatomotor network (SMN), default mode network (DMN), salience network (SN), dorsal attention network (DAN), precuneus network (PN), auditory network (AUN), executive control network (ECN), language network (LN), and frontoparietal network (PFN). The WFU_PickAtlas toolbox^2^ in the SPM toolbox, was utilized to create the RSN templates using radii and centroid coordinates. The component with the highest spatial correlation coefficient (*r* > 0.2) for each network was designated as the RSN of interest after identifying all ten components. The individual image maps for these components were subsequently used for second-level group analyses in SPM using two-sample *t*-tests. Statistical significance was determined at *p* < 0.05, family-wise error (FWE) corrected at the cluster level, with a voxel-wise threshold of *p* < 0.001 uncorrected applied to define clusters.

### 2.4 dFNC analysis

**Figure 2:**
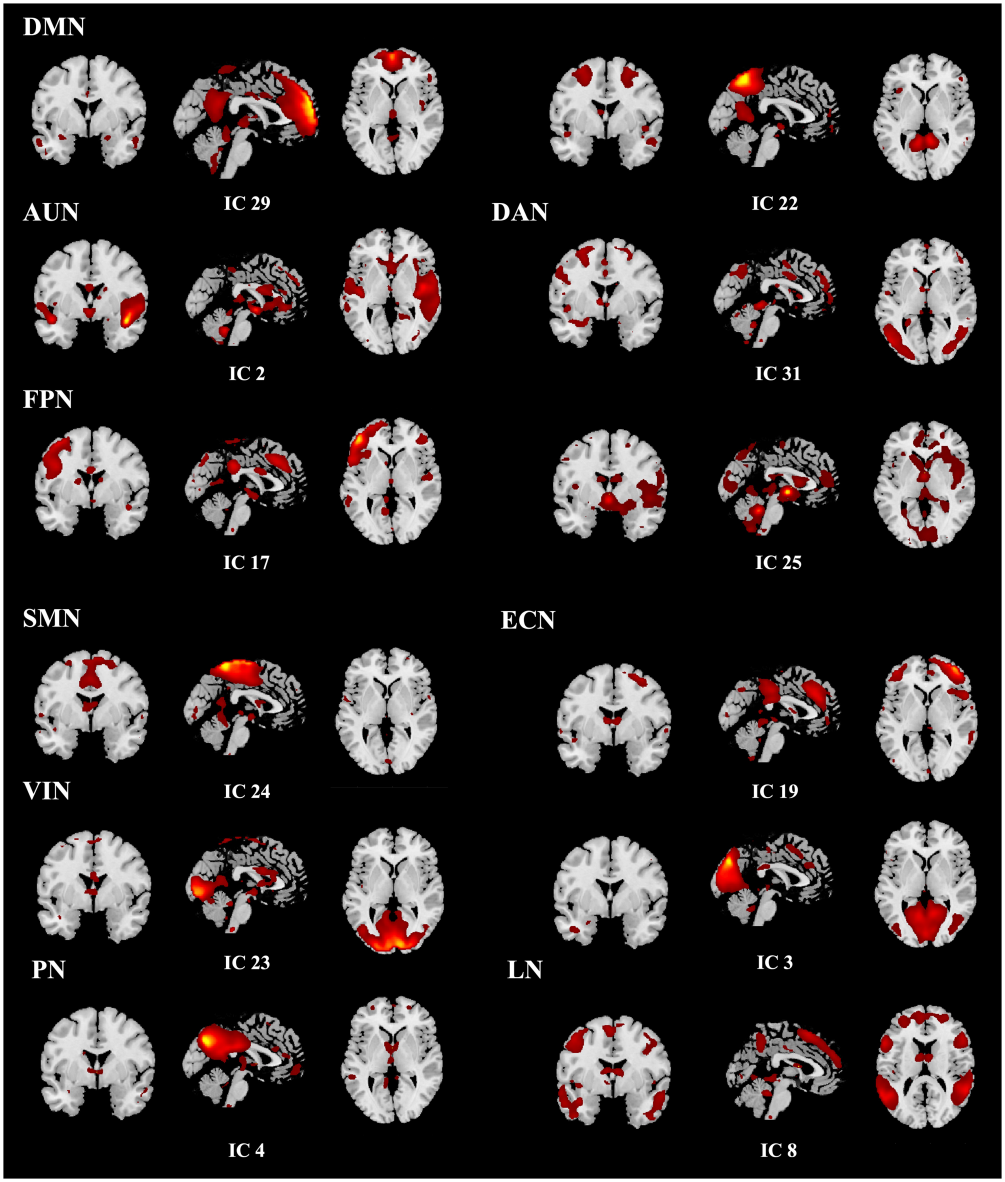
Spatial maps of the 12 selected resting-state networks, assigned to 9 functional domains.

For dFNC analysis, resting-state fMRI data from both HF patients and HCs were entered into a single group ICA using the GIFT toolbox. The number of independent components was estimated with the MDL criterion, which identified 31 components across the combined dataset. Following a similar approach to the previously described ICA procedure, components with the highest spatial correlation coefficients (*r* > 0.2), corresponding to the 10 previously identified RSNs, were selected. Ultimately, nine components were identified, with the SN (*r* = 0.167) excluded due to a lower correlation. The templates for the nine RSNs, including the corresponding ICs with which they showed the highest spatial correlations, are presented in Figure 2. The dFNC matrix was then computed using a sliding window method. Previous studies have suggested that window size ranging from 30–60 s can provide a robust estimation of the dynamic fluctuations in resting-state dFNC (24, 25). In this study, the window width was set to a TR of 30 (60 s), and the window was slid along the time axis in steps of 1 TR. The Pearson correlation coefficients between all pairs of BOLD signals in each window were calculated to construct a series of dynamic covariance matrices. Given that significant noise can influence the covariance estimates for short time series, L1 regularization (with 10 repetitions) was applied to improve the sparsity of each window’s dFNC matrix.

The k-means clustering algorithm was utilized to cluster the participants’ dFNC matrices to evaluate the structure and frequency of recurrent dFNC patterns. The Manhattan distance was then used to calculate how similar certain time windows were. To minimize the likelihood that a local minimum is reached during the clustering process, the iterations were set to a maximum of 500, with the process repeated 150 times. The elbow rule was used to find that k = 5 was the ideal number of clusters. All individuals’ dFNC matrices were categorized into five unique dFNC states, each indicating recurring instantaneous FC patterns across participants and windows. The dFNC matrix at each cluster’s center was identified in the present case as the cluster centroid.

The following information was also used for calculating several temporal features: (i) the reoccurrence fraction in each state (the percentage of total time the subject spent in each state); (ii) the mean dwell time which referred to the average duration a subject remained in a particular state, and (iii) the number of transitions which indicated how many times a subject switched between different states during the scan period.

### 2.5 Sample size determination

Sample size was estimated using G*Power 3.1 for two-sample *t*-tests. We assumed a medium-to-large effect size (d = 0.65), *α* = 0.05, and power = 0.80. This analysis indicated a required total of approximately 60 participants. Our final sample (HF = 35, HC = 28) therefore provided adequate statistical power for detecting effects of this magnitude.

### 2.6 Statistical analyses

Using the Stats module of the GIFT software, the dFNCs of the HF and HC groups were compared using two-sample *t*-tests with modifications for the false discovery rate (FDR). The threshold for statistical significance in this instance was set at *p* < 0.05. SPSS software 25.0 was used to evaluate the remaining data statistically. Normality was tested for continuous variables with the Kolmogorov–Smirnov test. Continuous variables were expressed as means ± SD and compared with two-sample *t*-tests for normally distributed data or the Mann–Whitney U-test for non-normally distributed data. Categorical group data presented as percentages were compared using the chi-squared test. Lastly, using age, sex, years of education, presence of diabetes and hypertension as control variables, Spearman’s partial correlation analysis investigated possible relationships between clinical variables (HAMA, HAMD, MMSE scores and LVEF) and dFNC temporal features. The Bonferroni correction was utilized with statistically significant differences at *p* × *n* < 0.05.

## 3 Results

### 3.1 Demographics and clinical characteristics

The HF and HC groups were comparable in age, sex, educational level, and prevalence of diabetes and hypertension (all *p* > 0.05). As expected, HF patients had markedly lower LVEF (*t* = 13.33, *p* < 0.001) and MMSE scores (*t* = 2.58, *p* = 0.011), and significantly higher HAMA (z = −9.133, *p* < 0.001) and HAMD (z = −8.817, *p* < 0.001) scores (Table 1).

### 3.2 Analysis of functional connectivity

**Figure 3:**
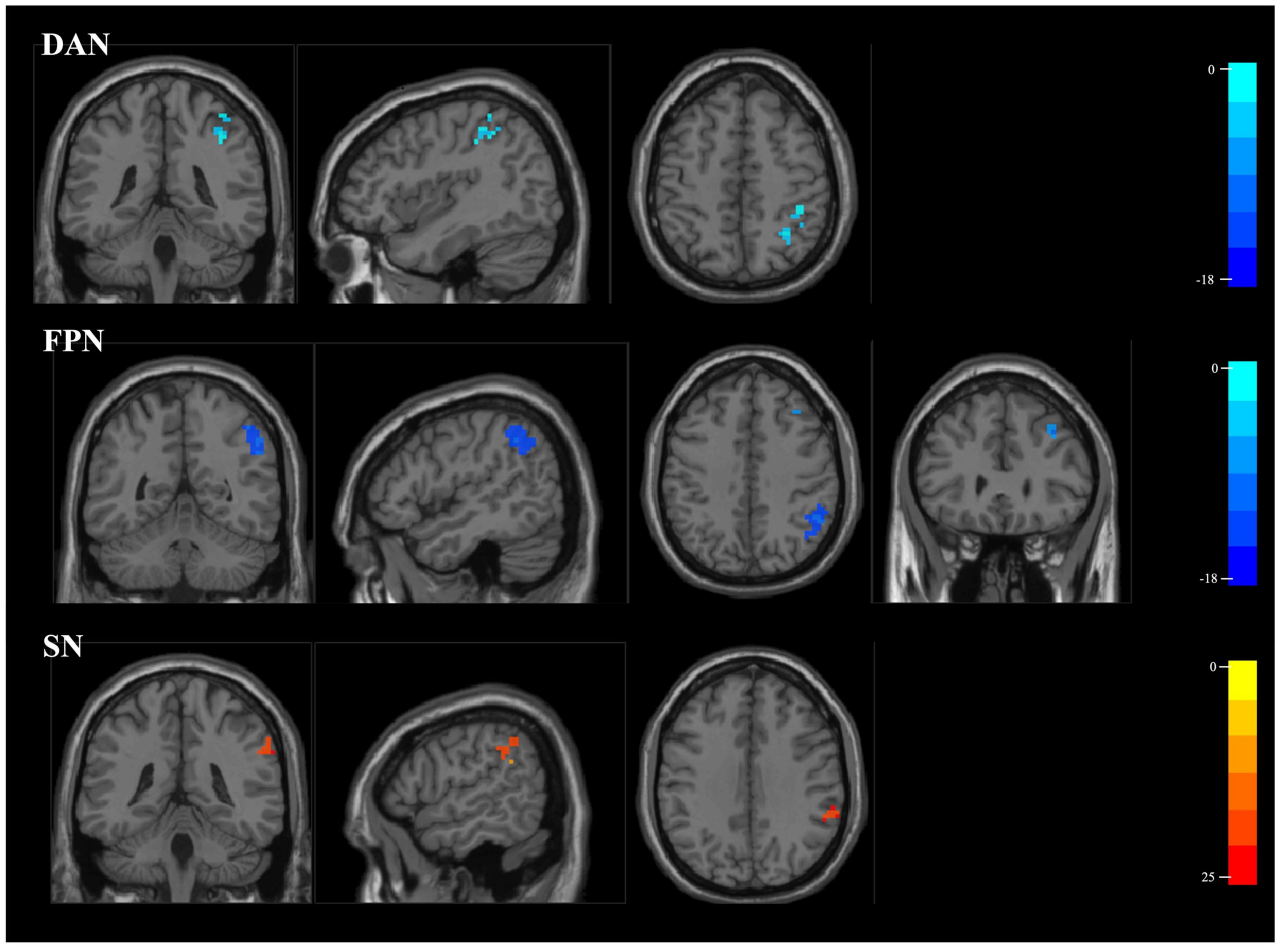
Results of ICA group comparison. Brain regions in HF patients exhibit decreased internal network FCs in the DAN and FPN, while showing significantly increased FC in the SN. The color bars indicate the *t* values of FCs. ICA, independent component analysis; HF, heart failure; FC, functional connectivity; DAN, dorsal attention network; FPN, frontoparietal network; SN, salience network.

The DAN, FPN and SN exhibited significantly different internal network FCs (*p* < 0.05, FEW corrected). Additionally, the FC in the right inferior parietal lobule (IPL) of the DAN was significantly reduced for HF patients (x = 39, y = −42, z = 45, cluster size = 29 voxels, x = 33, y = −54, z = 51, cluster size = 18 voxels), the right IPL, angular gyrus (AG), supramarginal gyrus (SMG) (x = 50, y = −51, z = 39, cluster size = 125 voxels), middle frontal gyrus (MFG) (x = 39, y = 36, z = 36, cluster size = 10 voxels) of FPN. Meanwhile, the HF patients exhibited a significant increase in the right SMG (x = 57, y = −39, z = 36, cluster size = 35 voxels) of SN (Table 2; Figure 3).

**Table 2:** Brain regions with significantly different FC values in the HF patients compared with the HCs.

HC > HF	Voxels	Peak MNI coordinates			Peak *t* value	pFWE	Brain region (voxels)
DAN	29	39	−42	45	6.13	0.000*	right IPL
	18	33	−54	51	6.24	0.000*	
FPN	125	50	−51	39	6.86	0.000*	right IPL (72) right AG (39) right SMG (14)
	10	39	36	36	5.39	0.002*	right MFG
HC < HF							
SN	35	57	−39	36	6.84	0.000*	right SMG

* FWE corrected, *p* < 0.05. HC, healthy control; HF, heart failure; DAN, dorsal attention network; PFN, frontoparietal network; SN, salience network; MNI, Montreal Neurological Institute; IPL, inferior parietal lobule; AG, angular gyrus; SMG, supramarginal gyrus; MFG, middle frontal gyrus.

### 3.3 Dynamic functional network connectivity states

**Figure 4:**
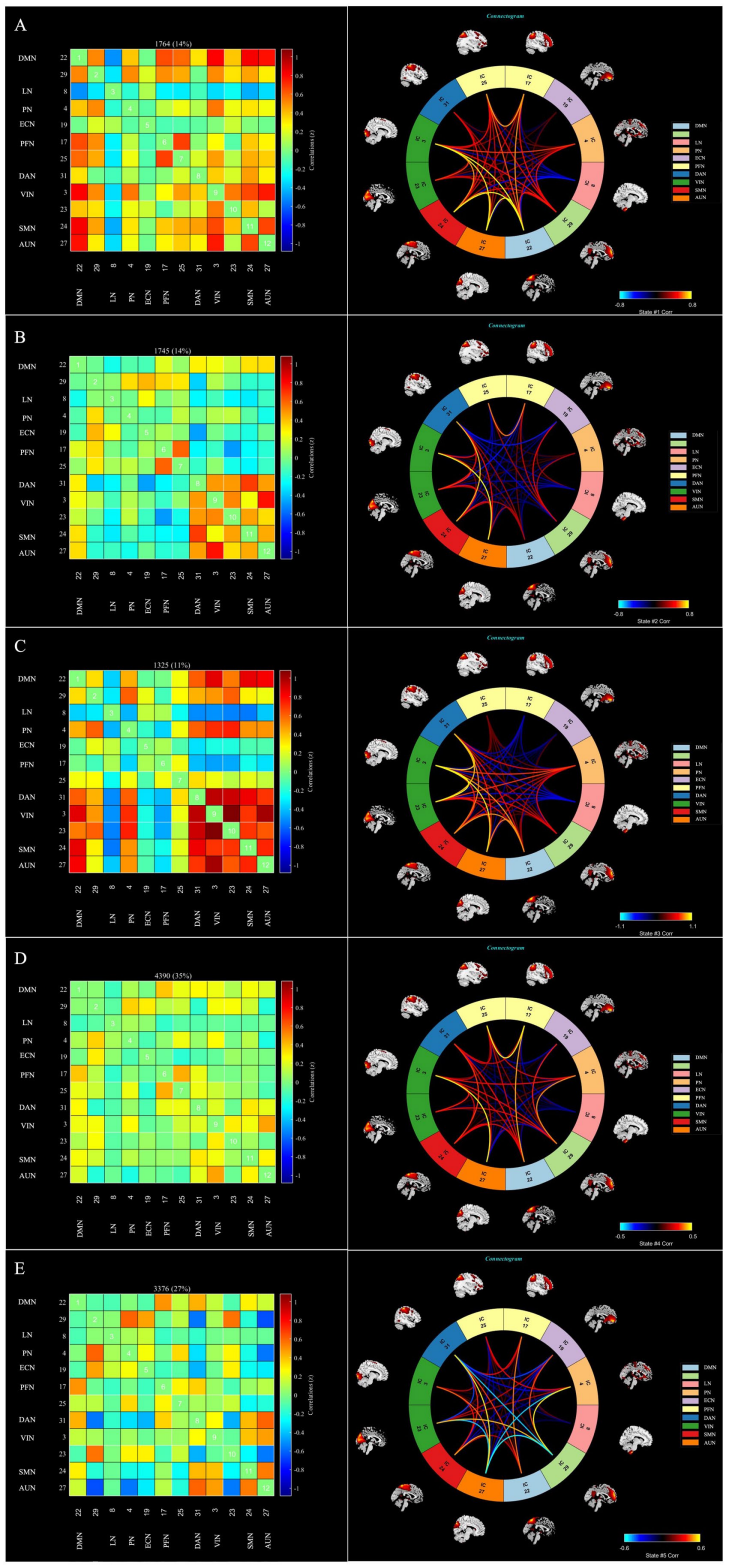
The dFNC matrices for state 1–5 and the dFNC diagrams for each state. The horizontal and vertical axes are the selected RSNs, and their functional networks are depicted. The color bars indicate the z values of the dFNC. dFNC, dynamic functional network connectivity; RSNs, Resting-state networks. The dFNC matrices for state 1–5 and the dFNC diagrams for each state **(A—E)**.

The following five recurring dFNC states were identified using the k-means clustering algorithm: state 1 (14% of all time windows), where higher cognitive control domains (DMN, FPN) and primary perceptional domains (AUN, VIN and SMN) showed a high level of positive inter-FNCs, with a high level of positively internal connectivity also noted within FPN. State 2 (14% of all time windows) where sparse FNCs were noted within and between all RSNs, except in the case of VIN, AUN, DAN and SMN, for which positive FNCs were found. State 3 (11% of all time windows) exhibited robust connectivity, particularly demonstrating significant positive connectivity among half-scale functional networks and negative connectivity in LN concerning the other networks. State 4 (35% of all time windows) showed sparse connectivity, with the functional network connectivity of the whole brain showing a general weakening. Lastly, State 5 (27% of all time windows), termed the sensory–cognitive segregation state, was characterized by negative coupling between sensory (VIN, AUN) and higher-order networks (DMN, PN), with preserved positive connectivity within higher-order systems (DMN–PN) and between attentional and auditory networks (DAN–AUN). The outcomes of the dFNC matrix index for each subject across all windows are displayed in Figure 4.

### 3.4 Temporal characteristics of states with functional connection

**Figure 5:**
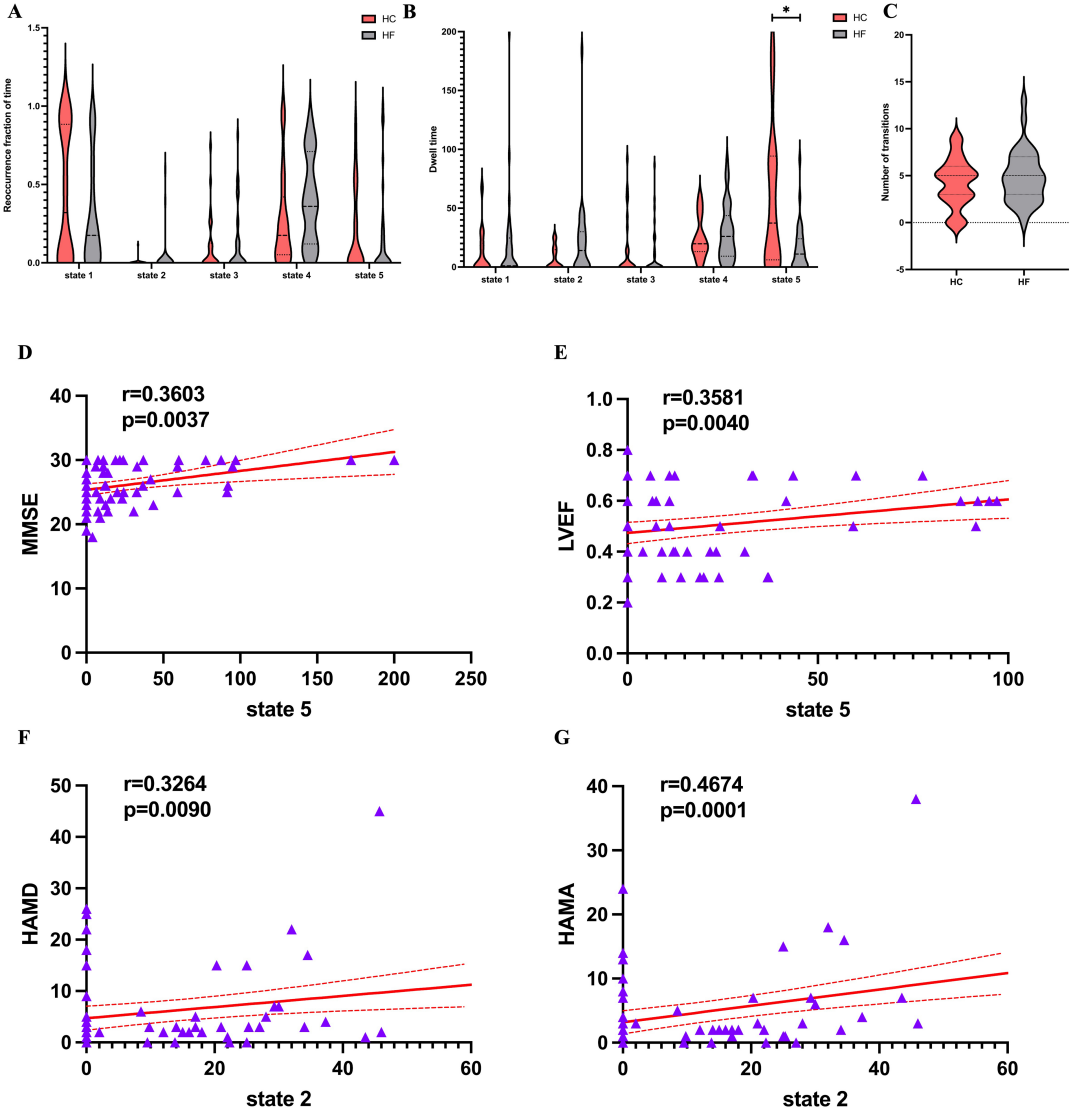
Temporal features of dFNC states for the HF and HC groups, and correlations between the temporal features and the clinical scales. **(A)** Reoccurrence fraction of time, **(B)** dwell time and **(C)** numbers of transitions were plotted using violin plots. Horizontal lines indicate group medians and interquartile ranges. The width of the violin plot bars indicates the distribution density of subjects in each group at the corresponding ordinate level. The horizontal lines above some violins indicate pairwise comparisons that demonstrated statistical significance: * *p* < 0.05. Cardiac function indicators. **(D,E)** A significantly positive relationship was observed between the mean dwell time in State 5 and the MMSE score (*r* = 0.360, *p* = 0.0037, Bonferrioni correction) and LVEF (*r* = 0.358, *p* = 0.004, Bonferrioni correction). **(F,G)** A significantly positive relationship was observed between the mean dwell time in State 2 and the HAMD score (*r* = 0.3264, *p* = 0.009, Bonferrioni correction) and HAMA (*r* = 0.467, *p* = 0.0001, Bonferrioni correction). dFNC, dynamic functional network connectivity; HF, heart failure; HC, healthy control; MMSE, Mini-Mental State Examination; LVEF, left ventricle ejection fraction; HAMD, Hamilton Depression Rating Scale; HAMA, Hamilton Anxiety Rating Scale.

The data proved that the mean dwell time was the longest for state 4, while state 3 had the shortest one. The former also appeared more frequently than state 2, which appeared least frequently. As shown in Figures 5A–C, the mean dwell time in state 5 was significantly lower for HF patients compared with HCs (Mann–Whitney U-tests, z = −2.63, *p* = 0.009 × 5 < 0.05 after Bonferroni correction), although in state 2, the HF patients showed an increasing trend in mean dwell time (Mann–Whitney U-tests, z = −2.056, *p* = 0.04 × 5 > 0.05 after Bonferroni correction). However, there was no discernible difference between the two groups for the fraction of time and number of transitions in any condition.

### 3.5 dFNC comparison between groups

Two-sample *t*-tests were used to compare the dFNC matrix of each state for the two groups. The HC and HF patients were not significantly different across states (FDR corrected, *p* < 0.05).

### 3.6 Correlation analyses

As shown in Figures 5D–G LVEF (*r* = 0.358, *p* = 0.004 × 5 < 0.05 after Bonferrioni correction) and MMSE (*r* = 0.360, *p* = 0.004 × 5 < 0.05 after Bonferrioni correction) were positive related to the mean dwell time of HF patients in state 5. The mean dwell time in state 2 showed a significantly positive connection with HAMD (*r* = 0.326, *p* = 0.009 × 5 < 0.05) and HAMA (*r* = 0.467, *p* < 0.001 × 5 < 0.05), even though the two groups did not differ significantly at state 2. Finally, clinical scales and cardiac function indicators were not significantly correlated with the remaining dFNC temporal metrics.

## 4 Discussion

Heart failure (HF) is increasingly viewed as a systemic condition in which impaired cardiac function disrupts brain integrity and network dynamics. The resulting cognitive decline and mood disturbances are not merely comorbidities but key determinants of prognosis, treatment adherence, and quality of life in affected patients. Accordingly, the European Society of Cardiology (ESC) and the American College of Cardiology/American Heart Association (ACC/AHA) currently emphasize the importance of screening HF patients for depression and cognitive impairment (23, 26). Although previous studies have highlighted structural and functional brain alterations in HF, less is known about the temporal dynamics of large-scale networks that may underlie these clinical manifestations. To address this gap, we applied group ICA and dynamic functional network connectivity (dFNC) analyses to compare HF patients with healthy controls. Our study yielded several key findings. At the static level, reduced intranetwork connectivity was observed in the DAN and FPN, particularly involving the right IPL, whereas in increased connectivity was detected within the SN, especially the right SMG. At the dynamic level, five recurring functional connectivity states were identified. Among these, the sensory–cognitive segregation state (State 5) exhibited significantly reduced dwell time in HF patients and was positively associated with both LVEF and MMSE scores. In contrast, the sparse connectivity state (State 2) showed prolonged dwell time in HF and was positively correlated with anxiety and depression scores.

We specifically focused on patients with LVEF < 50% (HFrEF and HFmrEF), as these subgroups share common pathophysiological features and are more likely to exhibit cognitive and network alterations related to reduced cardiac output. The static network findings highlight the IPL and MFG as critical regions affected in HF. The IPL, as a hub of both DAN and FPN, integrates sensory information (27, 28) and supports attention and episodic memory (29–33), and has also shown functional alterations in early-stage Alzheimer’s disease (AD) (34). Although we did not test a direct association between IPL connectivity and MMSE, the co-occurrence of reduced IPL connectivity and lower MMSE scores suggests that attentional and memory networks may be vulnerable in HF. The MFG, part of the dorsolateral prefrontal cortex, is central to executive control (35), and its dysfunction has been consistently linked to executive decline in mild cognitive impairment (MCI) (36). In contrast, the SN, particularly the SMG, functions as a dynamic hub for detecting salient stimuli (37) and coordinating inter-network switching (38). Increased SMG connectivity in HF may therefore represent a compensatory adaptation that helps sustain attention despite impaired DAN/FPN integrity. Altogether, reduced IPL and MFG connectivity, alongside compensatory SMG upregulation, point to early network-level alterations underlying cognitive vulnerability in HF, partly overlapping with mechanisms reported in MCI and AD, and may serve as potential imaging markers for cognitive monitoring and intervention in this population.

We employed dFNC to capture the temporal variability of large-scale network interactions (39). Five stable and recurrent dFNC states were identified across all subjects. Among them, State 4, characterized by sparse inter-network coupling, was the most predominant and exhibited the longest mean dwell time, likely reflecting baseline neuronal activity in the resting brain. State 5 was of particular interest, showing modular connectivity marked by negative coupling between sensory (AUN, VIN) and higher-order (DMN, PN) networks, alongside preserved positive coupling within higher-order systems. Given this pattern, we refer to it as the sensory–cognitive segregation state. In healthy individuals, prolonged engagement in this state may support the functional specialization of sensory and higher-order systems, enabling efficient information processing (40–42). By contrast, HF patients showed reduced dwell time, suggesting an impaired ability to maintain this modular configuration and a loss of balance between segregation and integration across large-scale networks. Such instability may reflect disruption of the heart–brain axis, whereby reduced cardiac output compromises the neural dynamics supporting sensory–cognitive interactions and cognitive performance. Consistent with this interpretation, shorter dwell time was associated with lower LVEF and MMSE scores, highlighting the link between impaired cardiac function and reduced stability of brain network dynamics (7, 43).

State 2, characterized by globally sparse connectivity with only limited positive coupling between sensory (VIN, AUN) and sensorimotor/attentional (SMN, DAN) networks, showed a trend toward longer dwell time in HF patients. Although the group difference was not significant, within HF patients dwell time in this state correlated positively with HAMA and HAMD scores. Prolonged engagement in a weakly connected, diffuse configuration may reflect inefficient interregional communication and heightened self-referential processing. Similar hypoconnected states have been reported in major depressive disorder, including altered connectivity between sensory and motor networks (44, 45). Such alterations may help explain the heightened susceptibility of HF patients to depression and anxiety.

### 4.1 Limitation

Firstly, this study was limited by a relatively small sample size, which may affect the generalizability and robustness of ICA/dFNC results. Nonetheless, significant group differences remained after stringent FWE/FDR corrections, suggesting robust effects. Future multi-center studies with larger cohorts are needed to replicate and extend these findings. Secondly, although detailed medication histories were collected for HF patients, prescriptions were highly heterogeneous and often irregularly used. With the relatively small sample size, it was not feasible to include medications as covariates; therefore, their potential confounding effects cannot be excluded. Future studies with larger and more homogeneous samples are warranted to better control for medication influences. Thirdly, this work did not exclude comorbidities in heart failure patients, such as hypertension/diabetes, and this can also lead to changes in brain FCs. However, the differences in these two conditions between the HF and HC groups were not statistically significant, thereby minimizing their potential influence. Fourthly, ECG gating and HRV-corrected reanalysis were not available, and although we applied rigorous preprocessing steps to mitigate physiological noise, residual cardiac- and respiration-related influences cannot be fully excluded; future multimodal studies incorporating concurrent physiological monitoring are warranted. Finally, cognition was assessed only with the MMSE, which may overlook subtle deficits; future studies should incorporate more sensitive tools such as the MoCA.

## 5 Conclusion

This study revealed altered static connectivity in IPL, MFG, and SN, together with dynamic abnormalities marked by reduced stability of a sensory–cognitive segregation state and prolonged engagement in a hypoconnected diffuse state. These network alterations were associated with cardiac function, cognition, and mood. We believe these findings provide novel neuroimaging evidence suggesting potential heart–brain interactions in HF, and may contribute to early identification of patients at risk for cognitive and emotional impairment.

## Data Availability

The raw data supporting the conclusions of this article will be made available by the authors, without undue reservation.

## Footnotes

^1^ http://rfmri.org/dpabi

^2^ https://www.nitrc.org/projects/wfu_pickatlas/

## References

[1] Savarese G Stolfo D Sinagra G Lund LH. Heart failure with mid-range or mildly reduced ejection fraction. Nat Rev Cardiol. (2022) 19:100–16. doi: 10.1038/s41569-021-00605-5, PMID: 34489589 PMC8420965

[2] Hammond CA Blades NJ Chaudhry SI Dodson JA Longstreth WT Jr Heckbert SR. Long-term cognitive decline after newly diagnosed heart failure: longitudinal analysis in the Chs (cardiovascular health study). Circ Heart Fail. (2018) 11:e004476. doi: 10.1161/circheartfailure.117.004476, PMID: 29523517 PMC6072263

[3] Doehner W Ural D Haeusler KG Čelutkienė J Bestetti R Cavusoglu Y. Heart and brain interaction in patients with heart failure: overview and proposal for a taxonomy. A position paper from the study group on heart and brain interaction of the heart failure association. Eur J Heart Fail. (2018) 20:199–215. doi: 10.1002/ejhf.1100, PMID: 29280256

[4] Celano CM Villegas AC Albanese AM Gaggin HK Huffman JC. Depression and anxiety in heart failure: a review. Harv Rev Psychiatry. (2018) 26:175–84. doi: 10.1097/hrp.0000000000000162, PMID: 29975336 PMC6042975

[5] Easton K Coventry P Lovell K Carter LA Deaton C. Prevalence and measurement of anxiety in samples of patients with heart failure: Meta-analysis. J Cardiovasc Nurs. (2016) 31:367–79. doi: 10.1097/jcn.0000000000000265, PMID: 25930162 PMC4915759

[6] Abou Kamar S Oostdijk B Andrzejczyk K Constantinescu A Caliskan K Akkerhuis KM. Temporal evolution of anxiety and depression in chronic heart failure and its association with clinical outcome. Int J Cardiol. (2024) 411:132274. doi: 10.1016/j.ijcard.2024.132274, PMID: 38880425

[7] Lee TC Qian M Liu Y Graham S Mann DL Nakanishi K. Cognitive decline over time in patients with systolic heart failure: insights from Warcef. JACC Heart Fail. (2019) 7:1042–53. doi: 10.1016/j.jchf.2019.09.003, PMID: 31779926 PMC6944056

[8] Sbolli M Fiuzat M Cani D O'Connor CM. Depression and heart failure: the lonely comorbidity. Eur J Heart Fail. (2020) 22:2007–17. doi: 10.1002/ejhf.1865, PMID: 32468714

[9] Chen J Normand SL Wang Y Krumholz HM. National and regional trends in heart failure hospitalization and mortality rates for Medicare beneficiaries, 1998-2008. JAMA. (2011) 306:1669–78. doi: 10.1001/jama.2011.1474, PMID: 22009099 PMC3688069

[10] Jung M Smith AB Giordani B Clark DG Gradus-Pizlo I Wierenga KL. Computerized cognitive training and 24-month mortality in heart failure. J Cardiovasc Nurs. (2024) 39:E51–8. doi: 10.1097/jcn.0000000000001023, PMID: 37494830 PMC10808269

[11] Beckmann CF DeLuca M Devlin JT Smith SM. Investigations into resting-state connectivity using independent component analysis. Philos Trans R Soc Lond Ser B Biol Sci. (2005) 360:1001–13. doi: 10.1098/rstb.2005.1634, PMID: 16087444 PMC1854918

[12] Wang Y Wang C Miao P Liu J Wei Y Wu L. An imbalance between functional segregation and integration in patients with pontine stroke: a dynamic functional network connectivity study. Neuroimage Clin. (2020) 28:102507. doi: 10.1016/j.nicl.2020.102507, PMID: 33395996 PMC7714678

[13] Hindriks R Adhikari MH Murayama Y Ganzetti M Mantini D Logothetis NK. Can sliding-window correlations reveal dynamic functional connectivity in resting-state Fmri? NeuroImage. (2016) 127:242–56. doi: 10.1016/j.neuroimage.2015.11.055, PMID: 26631813 PMC4758830

[14] Liu X Duyn JH. Time-varying functional network information extracted from brief instances of spontaneous brain activity. Proc Natl Acad Sci USA. (2013) 110:4392–7. doi: 10.1073/pnas.1216856110, PMID: 23440216 PMC3600481

[15] Cifre I Miller Flores MT Penalba L Ochab JK Chialvo DR. Revisiting nonlinear functional brain co-activations: directed, dynamic, and delayed. Front Neurosci. (2021) 15:700171. doi: 10.3389/fnins.2021.700171, PMID: 34712111 PMC8546168

[16] Mueller K Thiel F Beutner F Teren A Frisch S Ballarini T. Brain damage with heart failure: cardiac biomarker alterations and gray matter decline. Circ Res. (2020) 126:750–64. doi: 10.1161/circresaha.119.315813, PMID: 31969053 PMC7077969

[17] Bermudez C Kerley CI Ramadass K Farber-Eger EH Lin YC Kang H. Volumetric brain MRI signatures of heart failure with preserved ejection fraction in the setting of dementia. Magn Reson Imaging. (2024) 109:49–55. doi: 10.1016/j.mri.2024.02.016, PMID: 38430976 PMC11969415

[18] Mueller K Thiel F Taskin B Beutner F Teren A Dubovoy VK. Brain dysconnectivity with heart failure. Brain Commun. (2023) 5:fcad103. doi: 10.1093/braincomms/fcad103, PMID: 37091590 PMC10116577

[19] Zheng C Cui Y Gu S Yan S Cui B Song T. Cerebral hypometabolism mediates the effect of stroke volume on cognitive impairment in heart failure patients. ESC Heart Fail. (2024) 11:444–55. doi: 10.1002/ehf2.14599, PMID: 38037178 PMC10804188

[20] Park HJ Friston K. Structural and functional brain networks: from connections to cognition. Science. (2013) 342:1238411. doi: 10.1126/science.1238411, PMID: 24179229

[21] Jiang L Liu S Li L Wu W Ai Z Chen H. Aberrant static and dynamic functional network connectivity in heart failure with preserved ejection fraction. ESC Heart Fail. (2022) 9:2558–66. doi: 10.1002/ehf2.13967, PMID: 35560560 PMC9288811

[22] Cohen JR. The Behavioral and cognitive relevance of time-varying, dynamic changes in functional connectivity. NeuroImage. (2018) 180:515–25. doi: 10.1016/j.neuroimage.2017.09.036 28942061 PMC6056319

[23] Ponikowski P Voors AA Anker SD Bueno H Cleland JG Coats AJ. 2016 esc guidelines for the diagnosis and treatment of acute and chronic heart failure. Kardiol Pol. (2016) 74:1037–147. doi: 10.5603/kp.2016.0141, PMID: 27748494

[24] Fu Z Du Y Calhoun VD. The dynamic functional network connectivity analysis framework. Engineering (Beijing). (2019) 5:190–3. doi: 10.1016/j.eng.2018.10.001, PMID: 32489683 PMC7265753

[25] Savva AD Mitsis GD Matsopoulos GK. Assessment of dynamic functional connectivity in resting-state Fmri using the sliding window technique. Brain Behav. (2019) 9:e01255. doi: 10.1002/brb3.1255, PMID: 30884215 PMC6456784

[26] Heidenreich PA Bozkurt B Aguilar D Allen LA Byun JJ Colvin MM. 2022 Aha/Acc/Hfsa guideline for the Management of Heart Failure: a report of the American College of Cardiology/American Heart Association joint committee on clinical practice guidelines. Circulation. (2022) 145:e895–e1032. doi: 10.1161/cir.0000000000001063 35363499

[27] Spreng RN Sepulcre J Turner GR Stevens WD Schacter DL. Intrinsic architecture underlying the relations among the default, dorsal attention, and frontoparietal control networks of the human brain. J Cogn Neurosci. (2013) 25:74–86. doi: 10.1162/jocn_a_00281, PMID: 22905821 PMC3816715

[28] Gerlach KD Spreng RN Gilmore AW Schacter DL. Solving future problems: default network and executive activity associated with goal-directed mental simulations. NeuroImage. (2011) 55:1816–24. doi: 10.1016/j.neuroimage.2011.01.030, PMID: 21256228 PMC3855008

[29] Cabeza R Ciaramelli E Moscovitch M. Cognitive contributions of the ventral parietal cortex: an integrative theoretical account. Trends Cogn Sci. (2012) 16:338–52. doi: 10.1016/j.tics.2012.04.008, PMID: 22609315 PMC3367024

[30] Cabeza R Mazuz YS Stokes J Kragel JE Woldorff MG Ciaramelli E. Overlapping parietal activity in memory and perception: evidence for the attention to memory model. J Cogn Neurosci. (2011) 23:3209–17. doi: 10.1162/jocn_a_00065, PMID: 21568633 PMC3518433

[31] Igelström KM Graziano MSA. The inferior parietal lobule and temporoparietal junction: a network perspective. Neuropsychologia. (2017) 105:70–83. doi: 10.1016/j.neuropsychologia.2017.01.001, PMID: 28057458

[32] Zhan Y Ma J Alexander-Bloch AF Xu K Cui Y Feng Q. Longitudinal study of impaired intra- and inter-network brain connectivity in subjects at high risk for Alzheimer's disease. J Alzheimer's Dis. (2016) 52:913–27. doi: 10.3233/jad-160008, PMID: 27060962

[33] Jiang R Scheinost D Zuo N Wu J Qi S Liang Q. A neuroimaging signature of cognitive aging from whole-brain functional connectivity. Adv Sci (Weinh). (2022) 9:e2201621. doi: 10.1002/advs.202201621, PMID: 35811304 PMC9403648

[34] Wu H Song Y Yang X Chen S Ge H Yan Z. Functional and structural alterations of dorsal attention network in preclinical and early-stage Alzheimer's disease. CNS Neurosci Ther. (2023) 29:1512–24. doi: 10.1111/cns.14092, PMID: 36942514 PMC10173716

[35] Jones DT Graff-Radford J. Executive dysfunction and the prefrontal cortex. Continuum. (2021) 27:1586–601. doi: 10.1212/con.0000000000001009, PMID: 34881727

[36] Wang S Rao J Yue Y Xue C Hu G Qi W. Altered frequency-dependent brain activation and white matter integrity associated with cognition in characterizing preclinical Alzheimer's disease stages. Front Hum Neurosci. (2021) 15:625232. doi: 10.3389/fnhum.2021.625232, PMID: 33664660 PMC7921321

[37] Huang H Chen C Rong B Wan Q Chen J Liu Z. Resting-state functional connectivity of salience network in schizophrenia and depression. Sci Rep. (2022) 12:11204. doi: 10.1038/s41598-022-15489-9, PMID: 35778603 PMC9249853

[38] Verhulst M Keijzer HM van Gils PCW van Heugten CM Meijer FJA Tonino BAR. Functional connectivity in resting-state networks relates to short-term global cognitive functioning in cardiac arrest survivors. Hum Brain Mapp. (2024) 45:e26769. doi: 10.1002/hbm.26769 39449030 PMC11502408

[39] Rashid B Arbabshirani MR Damaraju E Cetin MS Miller R Pearlson GD. Classification of schizophrenia and bipolar patients using static and dynamic resting-state Fmri brain connectivity. NeuroImage. (2016) 134:645–57. doi: 10.1016/j.neuroimage.2016.04.051, PMID: 27118088 PMC4912868

[40] Smith R Thayer JF Khalsa SS Lane RD. The hierarchical basis of neurovisceral integration. Neurosci Biobehav Rev. (2017) 75:274–96. doi: 10.1016/j.neubiorev.2017.02.003, PMID: 28188890

[41] Petzschner FH Weber LA Wellstein KV Paolini G Do CT Stephan KE. Focus of attention modulates the heartbeat evoked potential. NeuroImage. (2019) 186:595–606. doi: 10.1016/j.neuroimage.2018.11.037, PMID: 30472370

[42] Tumati S Paulus MP Northoff G. Out-of-step: brain-heart desynchronization in anxiety disorders. Mol Psychiatry. (2021) 26:1726–37. doi: 10.1038/s41380-021-01029-w, PMID: 33504952

[43] Huijts M van Oostenbrugge RJ Duits A Burkard T Muzzarelli S Maeder MT. Cognitive impairment in heart failure: results from the trial of intensified versus standard medical therapy in elderly patients with congestive heart failure (time-Chf) randomized trial. Eur J Heart Fail. (2013) 15:699–707. doi: 10.1093/eurjhf/hft020, PMID: 23384944

[44] Yang W Wang Y Qin W Li M Mao H Zhou C. Abnormal dynamic functional network connectivity in first-episode, drug-naïve patients with major depressive disorder. J Affect Disord. (2022) 319:336–43. doi: 10.1016/j.jad.2022.08.072, PMID: 36084757

[45] Lu F Cui Q Huang X Li L Duan X Chen H. Anomalous intrinsic connectivity within and between visual and auditory networks in major depressive disorder. Prog Neuro-Psychopharmacol Biol Psychiatry. (2020) 100:109889. doi: 10.1016/j.pnpbp.2020.109889, PMID: 32067960

